# COVID-19-Driven Improvements and Innovations in Pharmacy Education: A Scoping Review

**DOI:** 10.3390/pharmacy10030060

**Published:** 2022-06-04

**Authors:** Jennifer Courtney, Erika Titus-Lay, Ashim Malhotra, Jeffrey Nehira, Islam Mohamed, Welly Mente, Uyen Le, Linda Buckley, Xiaodong Feng, Ruth Vinall

**Affiliations:** College of Pharmacy, California Northstate University, Elk Grove, CA 95757, USA; jennifer.courtney@cnsu.edu (J.C.); erika.titus-lay@cnsu.edu (E.T.-L.); ashim.malhotra@cnsu.edu (A.M.); jeffrey.nehira@cnsu.edu (J.N.); islam.mohamed@cnsu.edu (I.M.); wmente@cnsu.edu (W.M.); uyen.le@cnsu.edu (U.L.); linda.buckley@cnsu.edu (L.B.); xfeng@cnsu.edu (X.F.)

**Keywords:** pharmacy education, COVID-19, scoping review

## Abstract

The COVID-19 pandemic led to many colleges of pharmacy having to make major changes relating to their infrastructure and delivery of their curriculum within a very short time frame, including the transition of many components to an online setting. This scoping review sought to summarize what is known about the impact of COVID-19 on pharmacy education and the effectiveness of adaptation strategies which were put in place. PubMed, Web of Science, OVID Medline, and MedEdPortal were searched to identify pharmacy education-related articles published since the beginning of the COVID-19 pandemic. For article inclusion, the following criteria had to be met: described original research, related directly to PharmD or PharmBS education, related to the impact of COVID-19 on pharmacy education, and was available in English. Out of a total of 813 articles, 50 primary research articles were selected for inclusion. Our review of these identified four domains relating to the impact of COVID-19 on pharmacy education and/or effectiveness of adaptation strategies: (1) lab-based courses and activities (including interprofessional education activities), (2) experiential education, (3) didactic education, and (4) student well-being. The key research findings are summarized and discussed. While the COVID-19 pandemic has clearly brought many challenges to pharmacy education, it has also led to key improvements and innovations.

## 1. Introduction

The World Health Organization (WHO) officially characterized COVID-19 as a pandemic in March 2020 [1]. Soon after, many institutes of education throughout the world, including those housing pharmacy programs, had to stop some, if not all, in-person classes and either temporarily suspend their programs or transition to alternative modes to deliver their curriculum. These COVID-19-driven changes, many of which have been widely reported as reflection articles and opinion pieces, affected all stakeholders including students, faculty, staff, preceptors, and administrators as challenges were identified and addressed. The impact of COVID-19 on pharmacy education and specific stakeholders as well as the effectiveness of adaptation strategies has also been formally assessed through research studies. Often, new developments and innovations within education, including pharmacy education, are difficult to implement for various reasons, including a lack of buy-in from other faculty members and administrators, accreditation and student satisfaction concerns, and logistical reasons. The COVID-19 pandemic allowed these challenges to be overcome, and it is important to now reflect on what we can learn from what has been published. 

The majority of pharmacy programs transitioned to offering at least some of their curriculum via online platforms as part of their COVID-19-driven adaptation strategy. Undoubtedly, pharmacy programs that already offered online classes were better positioned to transition more of their curricular components to an online setting during the COVID-19 pandemic based on their existing infrastructure and experience. Several educational research studies published before the COVID-19 pandemic have shown that online courses are generally well accepted by pharmacy students and that academic standards can be maintained when pharmacy courses are offered online [2,3,4]. For example, our group and others have demonstrated that more than 50% of pharmacy students taking elective courses preferred online delivery and that student satisfaction levels with online elective courses are high [2,3,4]. Porter et al. directly compared online versus in-person delivery of the same didactic elective course and demonstrated that there was no difference in PharmD student performance levels based on course delivery method [3]. Research studies have also identified common challenges with online delivery, including decreased student engagement and concerns relating to the maintenance of exam integrity [5,6,7,8]. While these pre-COVID-19 studies support the use of online delivery within pharmacy education, they are limited in number and focus on primarily online delivery of didactic courses. The COVID-19 pandemic has allowed more research in this area; in particular, there is now a much stronger body of evidence describing online delivery of pharmacy lab-based classes and experiential education. More evidence relating to the impact of online delivery on pharmacy student well-being is also now available. 

The purpose of this scoping review was to summarize evidence relating to the impact of the COVID-19 pandemic on pharmacy education, as well as to highlight COVID-19-driven improvements and innovations. A review of educational research data and reflection on how the COVID-19 pandemic has impacted pharmacy education can help guide the future development of pharmacy programs and improve pharmacy education outcomes, as well as help inform contingency planning measures. 

## 2. Methods

### 2.1. Literature Search

A scoping search of the literature to find relevant articles was conducted using the search engines PubMed, Web of Science, OVID Medline, and MedEdPortal. Search terms related to pharmacy education and COVID-19 (details of the full search strategy can be found in the Supplementary Materials section). A citation chaining strategy was not employed.

### 2.2. Study Selection Criteria

Citations were imported into an Endnote library and duplicates removed. Two authors independently reviewed article titles, then article abstracts, and then the full text to identify articles for inclusion. At each step, a third author resolved any disagreements. For article inclusion, the following criteria had to be met: described original research, related directly to PharmD or PharmBS education, related to the impact of COVID-19 on pharmacy education, and was available in English. 

### 2.3. Article Review 

Following full-text review, the authors met to identify common themes (‘domains’) relating to pharmacy education. Articles were then assigned to one or more of these domains. The following characteristics for each study were also captured: year of publication, country where the study was conducted, number and type of participants, and study type. 

## 3. Results

**Figure 1:**
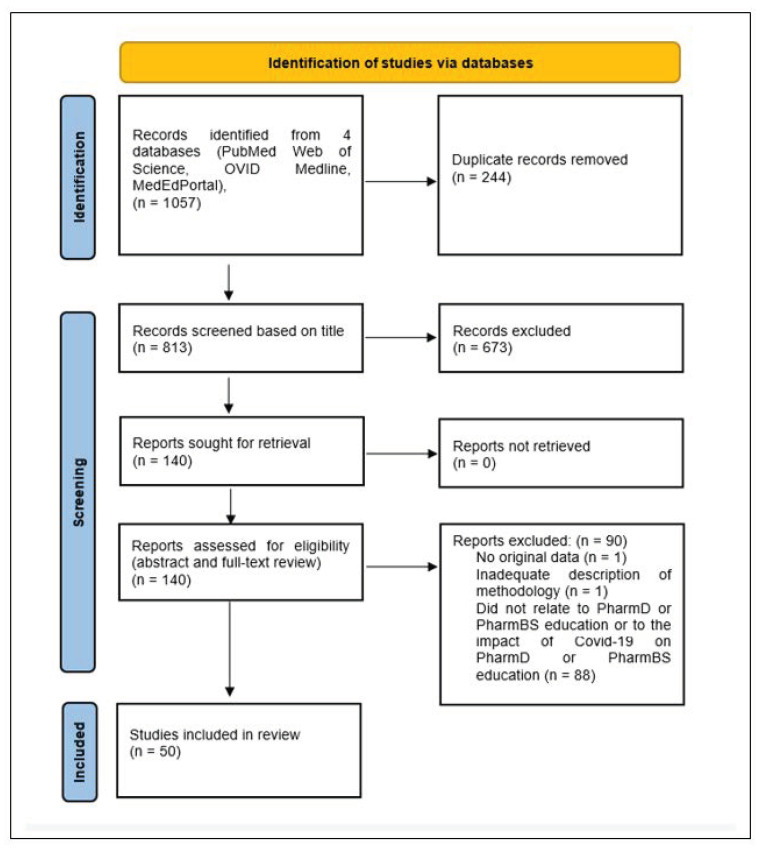
PRISMA flow chart for article inclusion.

Figure 1 shows the Preferred Reporting Items for Systematic Reviews and Meta-Analyses (PRISMA, http://www.prisma-statement.org/ (accessed on 10 January 2022)) flow chart for article inclusion. A total of 813 articles were identified from the literature search after removal of duplicates. After applying our inclusion strategy (article described original research, related directly to PharmD or PharmBS education, related to the impact of COVID-19 on pharmacy education, and was available in English), this was reduced to 50 articles. It is noteworthy that we did not have to exclude any articles due to their not being available in English. 

### 3.1. Characteristics of Selected Studies

We identified four main domains within the 50 articles selected for inclusion: impact of the COVID-19 pandemic on (1) lab-based courses and activities (including interprofessional education activities) (n = 17) (2) experiential education (n = 7) (3) didactic education (n = 17) and (4) student well-being (n = 14). Information for each of these studies, including year of publication, country where the study was conducted, number and type of participants, and study type, can be found in Table 1, Table 2, Table 3 and Table 4, along with an overview of the study results. 

### 3.2. Improvements and Innovations within Lab-Based Courses and Activities Which Allowed for Practice-Based Learning and Assessment of Student Competency to Continue during the COVID-19 Pandemic (n = 17 Studies)

Approximately 34% (17 out of 50 articles) of the included studies focused on the impact of COVID-19 on lab-based courses and activities, including Interprofessional Education (IPE) lab-based courses and activities (Table 1). Nine of the studies were conducted in the USA, three in Australia, two in Saudi Arabia, one in Malaysia, one in Turkey, and one in Germany. All of the studies included a survey analysis component, with 16 of the studies surveying student participants and one surveying administrators. Two of the studies assessed student course performance. Typically, surveys included Likert scale questions as well as free response questions followed by thematic analysis to identify key trends. Two of the survey studies included the implementation of a pre- post-test or conducting an end of course assessment within a given cohort as well as comparing data collected from pre-pandemic and pandemic cohorts. One study had a cross-over design study. 

Our review of these studies determined that multiple different strategies were employed to allow for the continuation of lab-based classes during the pandemic, including the use of face-to-face real-time video-conferencing, the generation and use of recorded videos to demonstrate key skill sets, requiring students to submit videos of themselves describing the skill or using role-play with family members/friends who are acting as mock patients to demonstrate competency, and the usage of MyDispense software. Overall, data from these studies indicate that virtual lab-based classes were well received by students, although several challenges were noted, including increased stress levels and technical challenges. The administrators who were surveyed also noted key challenges, including the inability to assess certain skills in an online setting and increased instructor stress levels [12]. Three of the studies focused on IPE-specific classes. The majority of students who participated in virtual IPE activities reported high levels of satisfaction and agreed or strongly agreed that activities helped clarified professional roles, improved communication and teamwork, and improved confidence levels [9,10,21]. 

Seven of the studies specifically surveyed students’ opinions relating to in-person versus virtual skills-based labs [13,15,16,18,19,20,23]. Three of the studies indicated that students felt less stressed during virtual lab simulations, while one study showed that although students had more time to prepare, most still felt an increase in stress levels during virtual skills-based lab simulations. Some of the advantages of virtual labs that were frequently noted by students included convenience, flexibility, and being easier to perform. In the article by Farahani et al., more than 95% of participants stated that more instructional videos should be included within all lab-based classes [24]. Three of the studies reported telehealth as a theme and noted the benefit and importance of being able to mimic emerging telehealth practices in pharmacy practice using web conferencing platforms [16,18,21]. Only one study focused on students’ opinions relating to virtual versus in-person learning after returning to in-person learning, and students in this study expressed a strong preference for in-person labs after having virtual labs the previous year [22]. This study also assessed the impact of returning to in-person lab courses on COVID-19 infection rates and transmission; no COVID-19 infections or transmissions were reported by students or their tracking system. To help minimize potential exposure and transmission, several infection control protocols and procedures were put into place; students were screened for COVID-19 symptoms, including a temperature check, prior to entering the building. Apps that track an individual’s exposure to COVID-19 were also used. Students were required to wear masks and were divided into smaller groups. Prior to lab classes, students were held in multiple areas, and social distancing was strictly implemented. Rooms were properly ventilated and simulated patients followed proper hand hygiene and social distancing norms, and students were asked to leave the building as soon as they finished the class. 

Two of the studies assessed the impact of offering virtual lab classes on course learning outcomes and scores by comparing student cohort performance in pre-pandemic in-person classes versus in virtual classes delivered during the pandemic classes [14,16]. These studies reported conflicting findings. The study by Aranda et al. found the students performed better on the pre-pandemic lab classes, while the study by Scoular found that students performed better on the virtual lab classes which were offered during the pandemic. The study by Scoular et al. noted concerns over exam integrity for the virtual lab classes [14]. 

Three articles described the use of commercially available simulation platforms [13,14,23]. Two of these used a virtual microbiology simulation (VUMIE), which simulated workflow in a microbiology wet lab [18,19]. Students were randomly split into two groups and either completed the wet lab activities or the VUMIE simulation. Both groups then completed the other activity not completed. This cross-over study design allowed for direct comparison of online versus in-person delivery within the same cohort of students simultaneously taking the same course. Analysis of student self-reporting of knowledge, skills, and confidence showed no difference between the in-person versus virtual experience; however, there was a strong preference for in-person labs. The other article described the use the ‘MyDispense’ platform, which simulates a virtual community pharmacy setting where students simulate pharmacy workflow and clinical decision-making skills [25]. A pre- and post-survey (42 questions) was given to assess student perceptions of confidence, satisfaction, motivation, expertise, and decision-making skills. The study data indicated a collective improvement in performance following participation in the virtual lab classes. It is noteworthy that the majority of students reported enhanced skills in clinical decision making and an improved ability to make drug therapy dispensing decisions. 

Two studies clearly showed benefit to using ‘MyDispense’ to help deliver lab-based classes and support its continued usage [25,58]. The ‘MyDispense’ software allows for practice with prescription processing, self-care recommendations, and patient assessment in an online setting. 

### 3.3. Improvements and Innovations within Experiential Education Which Supported Practice-Based Learning and Assessment of Professional Competency to Continue during the COVID-19 Pandemic (n = 7 Studies)

Approximately 14% (7 out of the 50 articles) of studies focused on the impact of COVID-19 on experiential education (Table 2). Five of the studies were conducted in the USA, one in Saudi Arabia, and one in Qatar. All of the studies were survey-based and only included student participants. Typically, surveys included Likert scale questions as well as free response questions followed by thematic analysis to identify key trends. Study designs included the implementation of a pre- post-test or conducting an end of course assessment within a given cohort, as well as comparing data collected from pre-pandemic and pandemic cohorts. One study reported average course grades for their virtual advanced pharmacy practice experience (APPE) rotation (although no comparator was included). 

All seven studies described the impact of offering virtual introductory pharmacy practice experience (IPPE) and APPE rotations and training. Overall, student experiences were positive and there was a high level of satisfaction with the materials presented. Most students felt that clear connections made between didactic knowledge and application of this knowledge within the training and that the virtual experiences helped them better understand relevance to pharmacy practice [26,27,28,29,30,31,32]. The collective data indicate that the most valuable aspects of the virtual APPE experiences were patient case presentations, journal clubs, topic discussions, and formal written assignments. Students did not rate written reflections as valuable as the other rotation assignments [26,27,28]. In the study by Montepara et al., students noted that they appreciated working with a variety of preceptors from different specialties and practice settings within the same virtual APPE rotation experience [28]. Common student concerns included anxiety about a lack of adequate patient care experience, and many students noted that virtual APPE activities should not completely replace in-person APPE rotations [26,31]. Suggestions for improvement included standardizing how information is disseminated to multiple students for virtual presentations and the virtual platforms used [28].

Reynolds et al. reported improvements in students’ perceptions of their performance as they relate to knowledge, skills, and abilities and calculations using a pre- post-course survey assessment, but these self-reported improvements were not statistically significant [30]. Overall course grades were high; however, no comparators were included. 

It is noteworthy that the study by Moreau et al. showed a telemedicine-based training experience that was well received by students [29]. As part of this study, students provided telephonic patient care service at the NSU College of Pharmacy’s call center. Students received training relating to medication adherence, medication therapy management, and transitions of care prior to starting their rotation. Most students agreed or strongly agreed that they gained a better understanding of the provision of pharmacy services through telehealth modalities as well as the use of value-based care models, including the utility of national quality benchmarks in this setting. Students also noted improvements in patient communication skills in this setting. 

### 3.4. COVID-19 Pandemic-Driven Improvements and Innovations in Online Delivery of Didactic Classes and in Online Testing (n = 17 Studies)

Approximately 34% (17 out of the 50 articles) of the included studies focused on the impact of COVID-19 on didactic classes (Table 3). One study was conducted in the USA, seven in Saudi Arabia, three in Jordan, two in Canada, one in Australia, one in Qatar, one in Malta, and there was one international-based study. The vast majority of studies (16 out of the 17) were survey-based, and most of these captured pharmacy student perspectives. Typically, surveys included Likert scale questions as well as free response questions followed by thematic analysis to identify key trends. One study assessed student performance. Study designs included the implementation of a pre- post-test or conducting an end of course assessment within a given cohort as well as comparing data collected from pre-pandemic and pandemic cohorts. 

Overall, based on data from these studies, the majority of pharmacy students appear to have had a positive experience with COVID-19-driven curricular changes relating to didactic classes (the most common change was transition to online delivery); however, there were some exceptions, and data from many studies indicate that students still prefer in-person classes. Frequently observed positive themes included ‘flexibility’ and ‘self-directed learning’. Frequently observed negative themes included ‘technology’, ‘study load’, and ‘well-being’. Only one study (Cor et al.) sought to determine the impact of COVID-19-driven changes on student performance. In this study, Hussain et al. compared midterm and final grades for a communications course offered pre-COVID-19 (offered via in-person delivery) versus offered during COVID-19 (offered via online delivery). No significant change in performance was observed. 

An innovation by Almaghaslah et al. was to use social media (Twitter and Instagram) to support the teaching of didactic classes during the pandemic [37]. Primarily, these platforms were used to complement formal teaching beyond core course hours. A survey conducted 4 weeks after the start of class showed that the majority of students ‘agreed’ or ‘strongly agreed’ that social media enhanced communication between peers and with instructors. This is important because well-being was a common theme reported in other studies. Use of the social media platforms was high (n = 73 for Twitter accounts, n = 69 for Instagram accounts); within the 4-week period, a total of 453 and 1740 ‘likes’ were reported on Twitter and Instagram, respectively, and the hashtag for the course was used 80 and 69 times in Twitter and Instagram posts.

Two studies included college of pharmacy administrators or faculty members as administrators [41,44]. The study by Alzubaidi et al. received survey responses from 46 college of pharmacy administrators and found that the majority felt that their institutions were able to offer adequate support to faculty members. Major challenges reported included ensuring exam integrity and the delivery of lab-based courses and experiential education [44]. Fifty-five percent of colleges reported disruption to their experiential program. In addition, administrators reported that many faculty members experienced significant levels of work-related stress, and some felt that their emotional support and training needs were not met. The study by Jaam et al. received survey responses from five faculty and found that faculty workload as well as testing integrity were key issues; however, as with the Alzubaidi et al. study, faculty members generally felt supported by their institutions [41]. 

### 3.5. Impact of the COVID-19 Pandemic on Student Well-Being; Lessons Learnt (n = 14 Studies)

Approximately 28% (14 out of the 50 articles) of the included studies focused on the impact of COVID-19 on student well-being (Table 4). Of the 14 studies that analyzed the emotional distress, anxiety, depression, or negative emotional impact of COVID-19, testing, and examination during COVID-19, 35.7% (5 out of 14) were cross-sectional in design, with a majority of the studies, 57.1% (8 of 14), conducted in pharmacy programs outside of the United States. Of these eight international programs, five were located in the Middle East, and one each was located in Canada, Spain, and Australia. Four programs (28.6%) were listed as undergraduate pharmacy programs, and one specifically mentioned analysis of emotional and mental health concerns for pharmacy graduate students and included pharmacy students who were also working towards earning a Ph.D. Almost all studies except two (85.7%) used some form of a perception student questionnaire or survey to collect data. The remaining two studies used an interview format followed by codification and identification of the themes related to COVID-19 emotional distress. Many of the studies compared student stress levels before and during the COVID-19 pandemic.

While 13 of the 14 (92.3%) included studies reported some form of a negative impact of either COVID-19 or the adaptation of online learning such as increases in stress and anxiety levels, one study (0.07%) reported a decrease in student stress levels before exams. Specifically, Hettinger et al. reported on the impact of COVID-19 on pharmacy student stress during high-stakes performance-based assessments before and during COVID-19 [11]. Based on data collected from responses from students at the Purdue University School of Pharmacy, stress levels before the performance-based exam fell from 3.78 to 3.45 when comparing pre-COVID-19 to during COVID-19, while stress levels similarly decreased from 2.84 to 2.52 after performance-based exams when comparing pre-COVID-19 to during COVID-19. It is noteworthy that Purdue College of Pharmacy hired a wellness officer during the pandemic to integrate wellness activities into the curriculum, and doing so most likely helped mitigate COVID-19-driven increases in student stress levels. 

Three studies (21.4%) analyzed the effect of COVID-19 on student quality of life, with specific references to stress, anxiety, and depression, while another three studies (21.4%) included coping strategies and resiliency mechanisms as part of the student survey instruments and measured the impact of coping strategies on emotional distress. Only one of the fourteen studies (0.07%), led by Moreno-Fernandez et al., included emotional intelligence as a part of the student response paradigm and found that inclusion of an emotional intelligence workshop alleviated self-reported academic burnout from 63.5% before to 31.1% after the emotional intelligence workshop [57]. They also reported high levels of stress and negativity in pharmacy students in Spain during COVID-19 who responded to the survey (n = 47), with 44.6% of the respondents reporting exhaustion, 41.7% reporting cynicism, and 60.3% reporting ineffective academic performance due to COVID-19. 

Interestingly, Atarabeen et al. included an analysis of pharmacy students in a program in the United States that employs flipped classroom pedagogy [48]. This is significant since flipped classrooms place significant emphasis on pre-classroom work, and thus, students may be expected to experience higher stress and anxiety levels. These investigators analyzed the impact of COVID-19 and the consequent adaptation to online and remote delivery on students’ stress, coping behaviors, self-efficacy, and emotional status. Of the 66 students who responded, interestingly, no significant differences were found between any of these four categories before and during COVID-19. 

## 4. Discussion

This scoping review summarizes data from research studies which sought to determine the impact of the COVID-19 pandemic on pharmacy education. Our review of these studies has identified common trends as well as areas which would benefit from further research. We also highlight key COVID-19-driven innovations and improvements which were found to be and/or perceived to be effective. 

The vast majority of studies focused on students rather than faculty members or administrators, and many were survey-based. Most focus was placed on assessing student perceptions of COVID-19-driven changes and their effectiveness. Only a few studies included an assessment of the impact of COVID-19-driven changes on student scores and grades. Implementation of more outcomes-based studies are needed to further establish the effectiveness of virtual classes and experiences and thereby help guide decisions regarding their continued usage beyond the pandemic. Typically, surveys comprised a combination of Likert score and open-ended questions; the latter allowed for thematic analysis and for the identification of key benefits as well as issues and concerns. Several study designs were employed for survey studies, including conducting a pre- post-test or survey within a given class or course or simply conducting an end of course survey. Several studies compared data collected prior to the pandemic with pandemic data, and while useful, these data should be interpreted with caution due to potential confounding effects. Only one study used a cross-over design which allowed for the intervention to be assessed within the same cohort. 

The most frequently described COVID-19-driven change was the transition of classes and experiences to an online setting, with many studies reporting the use of virtual platforms such as Zoom, BigBlueButtom, and Microsoft Teams. MyDispense software was found to be helpful for the delivery of lab-based classes and for rotations. Data from the majority of the studies indicate that most pharmacy students were satisfied with the online delivery of didactic classes, lab-based classes, and practice experiences, although most students stated they still preferred in-person delivery. This finding aligns with what has been reported by other medical education programs; for example, a survey study by Stoeher et al. (n = 3286 students from 12 medical schools) found that the majority of students were satisfied with the transition to online learning [59]. 

Many of the 50 studies included in this scoping review conducted thematic analyses, and these identified ‘flexibility’ and ‘self-directed learning’ as benefits to online learning and identified ‘cheating’, ‘mental health’, ‘communication’, ‘patient care experience’, and ‘technical issues’ as key challenges. Increasing and supporting self-directed learning experiences aligns well with directives from pharmacy governing bodies such the Accreditation Council for Pharmacy Education (ACPE) and is a key competency for any healthcare professional including pharmacists. It is therefore encouraging that students identified this as a positive associated with online delivery, and in this regard, the data support the continued usage of online delivery for at least some components of pharmacy education. Cheating on exams is well known to be a challenge associated with online delivery of any higher education course or experience, and this has been reported in both pre- and post-COVID-19 studies [5,6,7,8]. None of the studies included in this scoping review specifically focused on this aspect or how to address it; however, there are many opinion pieces and commentaries available describing potential solutions, and an increasing number of studies relating to this topic have been published in other areas of higher education, including other professional programs [60,61,62,63,64]. Further research on this topic would be beneficial to help support the continued usage of online delivery of didactic and lab-based courses as well as virtual rotation experiences in pharmacy education. The collective data also support the development of strategies to assess and support student well-being and mental health as part of contingency planning and align with what has been reported by other institutions of education including medical education programs. For example, a survey study by Chaklader et al. (n = 300 from 5 medical schools) found that students reported higher levels of depression and anxiety during the pandemic [65]. It is noteworthy that the one study which showed student stress levels were reduced during the pandemic reported hiring a wellness officer to integrate wellness activities into their curriculum [11]. The positive effects on student wellness reported in this study support this as an effective strategy for other colleges of pharmacy as well as other institutes of education to consider. 

The successful use of online delivery of didactic classes pre-pandemic and during the pandemic is well described in the literature for many types of higher education, including pharmacy education, with many students indicating high levels of satisfaction with online education [2,3,4,5,6,7,8,66]. As mentioned above, it would be helpful for future studies in pharmacy education to place more focus on the impact of online learning on student learning outcomes (in this scoping review, only one study assessed course scores and grades and showed no difference in performance). A key innovation described by Almaghaslah et al. was the use of social media platforms (Twitter and Instagram) to support extracurricular activities during the pandemic, including use of these to facilitate group discussion of practice questions and class discussions [37]. A systematic review of medical education during the COVID-19 pandemic revealed that multiple medical programs also successfully used social media-based learning strategies to help engage students [67]. 

The delivery of lab-based classes during the COVID-19 pandemic was extremely challenging for many reasons, including the need to deliver additional content relating to the COVID-19 pandemic-driven expansion in pharmacist scope of practice, as well as the difficulties associated with accommodating the meaningful assessment of lab-based skills in an online setting. A major benefit that was reported by several studies, and which would support continued usage of online learning activities, was their ability to help develop and support the use of skills relating to telemedicine, a field that is rapidly expanding. Similar findings have been reported within medical education [68,69]. Again, increased focus on how online delivery of lab-based classes impacts student course performance is needed to help drive continued usage of many of the strategies described. It is noteworthy that one of the studies reported being able to safely offer lab-based classes in person through the development and implementation of strict infection control protocols [22]. This, along with other data from the other studies which demonstrate that students clearly prefer in-person lab-based classes, support the expanded development of similar infection control policies and procedures within contingency plan measures. 

The continuation of IPPE and APPE rotations during the COVID-19 pandemic was also extremely challenging. As with the other curricular areas, a key strategy was the implementation of virtual IPPE and APPE rotations. The successful use of virtual clinical rotations during the COVID-19 pandemic has been reported by several medical schools [70,71,72,73], and the combined data indicate that continued usage of virtual rotations is a viable option. As noted above, telemedicine is a growing field in pharmacy practice, and the data indicate that increased offering of telemedicine experiences would be welcomed by students. A common student concern was the lack of adequate patient care experience, and the data indicate that in-person rotations are important and should not be completely replaced by virtual experiences. This domain had the fewest number of studies available, and many of them had a low number of participants. Only one study reported assessment data and no comparator was included. Clearly, there is a need to conduct more studies in this area to help guide the development of effective virtual rotation experiences, with a focus on telemedicine. 

## 5. Study Limitations

While a PRISMA-based strategy was used to identify the studies reported in this scoping review, it is possible that not all relevant studies were included. Many of the studies reported here have a relatively small sample size and not all studies include specific details regarding recruitment of participants. Based on this limitation, the presence of study bias cannot be ruled out. It is also noteworthy that many studies were more focused on proof of concept rather rigorous assessment, which is a reflection of the relativeness newness of this area of research. 

## 6. Conclusions

Pharmacy educators and administrators have gone above and beyond to help ensure student success throughout the COVID-19 pandemic, and many have leveraged this crisis to further improve pharmacy education. There is clearly a need for more focus on assessment of student performance outcomes in future research studies. In addition, more focus is needed on the impact and effectiveness of virtual experiential rotations on professional development and assessment of the impact of COVID-19 on pharmacy educators (only two of the fifty articles collected data from faculty members and/or administrators). The collective survey data indicate that the majority of students were satisfied with the COVID-19-driven strategies described; however, it is clear that contingency planning should place focus on student wellness and that there is a need to develop improved strategies to minimize cheating in online assessments. The development of infection control policies and procedures should also be considered within contingency planning to help enable more classes, particularly lab-based classes, to continue in person (based on data which support that infection control policies and procedures can be effective, as well as the clear student preference for in-person learning). Key improvements and innovations include increased usage of telemedicine and increased training that supports development of telemedicine-related skills. COVID-19 has served as a key disruptor to pharmacy education, and it is likely that COVID-19-driven improvements and innovations will continue to occur and be put into wider use based on the sharing and discussion of experiences that have been reported within the pharmacy education community and beyond.

## Figures and Tables

**Table 1:** Domain 1, lab-based courses activities including interprofessional education (IPE) activities (n = 17).

First Author, Year, Country	Study Objectives: Topic/Intervention/Innovation	Number/Type of Participants	Study Design	Brief Summary of Study Results
1. Bautista, 2020, USA [9]	Determine whether virtual IPE activities improve student confidence in meeting course learning objectives, assessment of perceived course quality	5 pharmacy students	End of class/activity survey, Likert scale and open-ended questions followed by thematic analysis, no control/comparator group included	The virtual IPE experience was well received by students and deemed to be effective, it increased students’ knowledge of professional roles
2. Martinez, 2021, USA [10]	Determine impact of a virtual IPE activity on student confidence levels	30, pharmacy students	Pre- post-class/activity survey, Likert scale questions	Virtual IPE activity improved student confidence levels
3. Hettinger, 2022, USA [11]	To compare stress levels of students taking skills-based assessment before/after the pandemic	801 pharmacy students (426 pre-pandemic, 375 post-pandemic)	Survey study, pre- versus post pandemic cohort comparison (data collected in different years), end of class/activity survey, open-ended questions followed by thematic analysis.	Students experienced higher levels of stress during the pandemic
4. Nolan, 2021, USA [12]	Determination of if/how colleges of pharmacy were assessing clinical skills during the pandemic	Administrators from 10 colleges of pharmacy	Likert scale survey followed by post-survey interview with open-ended questions and thematic review, no control/comparator group included	Most colleges continued skills assessments, challenges included lack of time to prepare and inability to assess some skills virtually
5. Savage, 2021, USA [13]	Identification of likes, dislikes, learning experience, and suggestions improvements for virtual OSCEs	156 pharmacy students	End of class/activity survey, open-ended questions followed by thematic analysis, no control/comparator group included	Overall, virtual OSCEs were well received and deemed application to their future pharmacy practice
6. Scoular, 2021, USA [14]	Determination of whether there are differences in performance levels between in-person versus virtual OSCEs	250 pharmacy students (144 in-person, 106 virtual)	Course performance assessment, comparison of in-person versus online learning (same cohort but data collected in different years)	Scores were higher for virtual versus in-person OSCEs
7. Thomas, 2022, USA [15]	Evaluation of students’ perceptions of an online pharmacogenomics lab activity	31 pharmacy students	End of class/activity survey, Likert scale and open-ended questions followed by thematic analysis, no control/comparator group included	The majority of students preferred online labs; students learned how to use pharmacogenomics databases
8. Aranda, 2020, USA [16]	Assess student performance, perceptions of a virtual physical exam OSCE	95 pharmacy students	End of class/activity survey, Likert scale questions, no control/comparator group included	Students performed better in in-person classes but still preferred a blended learning format over in-person classes
9. Wilhite, 2021, USA [17]	Comparison of student performance in a simulation-based lab course offered online versus in-person	264 pharmacy students	Course performance assessment for students taking in-person versus virtual classes (same cohort, same year); comparison of passing rates for course components and number of remediations	Performance levels were similar for students in online versus in-person courses
10. Baumann-Birkbeck, 2021, Australia [18]	Compare impact of in-person versus virtual microbiology labs on student knowledge, skills, and confidence	124 pharmacy students	Randomized crossover study within the same cohort, pre- post-survey self-reporting of perceived knowledge, skills, and confidence/Likert scale questions	No reported difference in outcomes between in-person versus virtual lab
11. Baumann-Birkbeck, 2022, Australia [19]	Assess students’ attitudes towards a virtual microbiology lab	39 pharmacy students	Randomized crossover study within the same cohort, pre- post-survey, Likert scale questions	Students found the virtual lab valuable, reported a preference for in-person labs
12. Mak, 2022, Australia [20]	Evaluation of student experiences with virtual OSCEs	190 pharmacy students	End of class/activity survey, open-ended questions followed by thematic analysis, no control/comparator group included	Sixty-seven percent of participants preferred in-person OSCEs, identified lack of non-verbal communication as a barrier to using virtual OSCEs
13. Alrasheed, 2021, Saudi Arabia [21]	Exploration of the benefits and limitations of virtual IPE activities	27 pharmacy students	End of class/activity focus group, open-ended questions followed by thematic analysis, no control/comparator group included	Most students valued the virtual IPE experience, students felt it clarified their roles and improved communication and teamwork skills
14. Alshaya, 2021, Saudi Arabia [22]	Determine whether in-person OSCEs increased COVID-19 transmission rates, assess student satisfaction	184 pharmacy students	Survey study, online delivery versus in-person delivery cohort comparison (data collected in different years), end of class/activity survey, Likert scale questions. Assessment of COVID-19 incidence and transmission, no control/comparator group included	No reported cases or transmission of COVID-19, increased student satisfaction levels with in-person classes
15. Elnaem, 2021, Malaysia [23]	Assess student perceptions of virtual OSCEs	253 pharmacy students	End of class/activity survey, Likert scale and open-ended questions followed by thematic analysis, no control/comparator group included	Students were satisfied with virtual OSCEs but still preferred in-person OSCEs
16. Farahani, 2021, Germany [24]	Assess student perceptions and satisfaction with an educational video for blood pressure measurement training	37 pharmacy students	End of class/activity survey, Likert scale and open-ended questions followed by thematic analysis, no control/comparator group included	More than 95% of participants stated instructional videos should be included in pharmacy education
17. Aksoy, 2021, Turkey [25]	Determine how use of MyDispense affected student learning outcomes, satisfaction	81 pharmacy students	Pre- post-class/activity survey, Likert scale questions	MyDispense activity was well received, improved knowledge and skills

**Table 2:** Domain 2, experiential education (n = 7).

First Author, Year, Country	Study Objectives: Topic/Intervention/Innovation	Number and Type of Study Participants	Study Design	Study Results
1. Johnston, 2021, USA [26]	Evaluate student perceptions of virtual APPEs	19 pharmacy students	End of class/activity survey, Likert scale and open-ended questions followed by thematic analysis, no control/comparator group included	High levels of satisfaction reported but stated virtual APPEs should not completely replace in-person experiences
2. Kiles, 2021, USA [27]	Describe student experiences with a remote public health APPE	16 pharmacy students	End of class/activity survey, Likert scale questions, no control/comparator group included	Student ratings were positive
3. Montepara, 2021, USA [28]	Examine student experiences with virtual APPE training	21 pharmacy students	End of class/activity survey, Likert scale questions, no control/comparator group included	The majority of students reported a positive experience, noted the patient case discussions were very helpful
4. Moreau, 2022, USA [29]	Examine students’ perception of a didactic-experiential telehealth elective course	6 pharmacy students	End of class/activity survey, Likert scale questions, no control/comparator group included	Most students agreed that they gained a better understanding of telehealthcare
5. Reynolds, 2021, USA [30]	Examine the perceived effectiveness of virtual IPPE	6 pharmacy students	Course performance assessment (no comparator), end of course survey, Likert scale questions, no control/comparator group included	Students agreed that the experience was valuable, relevant to pharmacy practice, student performance levels were high
6. Almohammed, 2021, Saudi Arabia [31]	Examine student experiences with virtual APPE training	87 pharmacy students	End of class/activity survey, Likert scale and open-ended questions followed by thematic analysis, no control/comparator group included	Student experiences were mostly positive, students enjoyed flexibility but were anxious about lack of adequate patient care experience
7. Al-Naimi, 2020, Qatar [32]	Examine student leader perceptions of pharmacy education during the pandemic	5 pharmacy students	Cross-sectional survey study, open-ended questions followed by thematic analysis, no control/comparator group included	Postponement of experiential learning was noted as a key challenge

Advanced pharmacy practice experience (APPE), Introductory pharmacy practice experience (IPPE).

**Table 3:** Domain 3, didactic courses (n = 17).

First Author, Year, Country	Study Objectives: Topic/Intervention/Innovation	Number of PARTICIPANTS *	Study Design	Study Results
1. Al-Neklawy, 2022, Saudi Arabia [33]	Evaluate the impact of online TBL on student perceptions of recall, engagement, and satisfaction	25 pharmacy students	End of class/activity survey, Likert scale questions, no control/comparator group included	The majority of students had a positive experience relating to impact on recall, engagement, and overall satisfaction
2. Alghamdi, 2021, Saudi Arabia [34]	Examine student experiences with online education	241 pharmacy students	End of class/activity survey, Likert scale and open-ended questions followed by thematic analysis, no control/comparator group included	Majority of students noted no negative impact relating to the transition to online education
3. Ali, 2021, Saudi Arabia [35]	Exploration of students’ perceptions of COVID-19 on their learning	790 pharmacy students	Thematic review of a survey conducted via twitter, open ended questions, no control/comparator group included	Key themes identified included; facilitators, barriers, online versus onsite, long-term impact, suggestions for improvement
4. Ahmed, 2021, Saudi Arabia [36]	Examination of perceptions of students towards online learning	50 pharmacy students	Survey study, Likert scale and multiple choice questions, no control/comparator group included	The majority of students prefer in-person classes, nearly half of students had a positive experience with online learning
5. Almaghaslah, 2021, Saudi Arabia [37]	Comparison of different media platforms to support online learning	67 pharmacy students	Capture of number of likes, comments on Twitter and Instagram relating to pharmacy education posts, post course survey study with Likert scale questions, no control/comparator group included	LMS (Blackboard) was preferred for academic use, students found use of social media platforms helpful for delivery of extracurricular material and for class discussions
6. Alqurshi, 2020, Saudi Arabia [38]	Investigate the effect of emergency remote teaching on pharmacy education	700 pharmacy students	Cross-sectional survey, Likert scale questions, no control/comparator group included	Almost half of students were concerned by the lack of guidance provided for online assessments
7. Shawaqfeh, 2020, Saudi Arabia [7]	Examination of the distance learning experience	309 pharmacy students	Cross-sectional survey study, Likert scale and multiple choice questions, no control/comparator group included	The majority of students felt adequately prepared for online learning and had a positive experience, one third of participants identified challenges
8. Elsalem, 2021, Jordan [39]	Evaluation of students preference for remote versus in-person exams, academic dishonesty	84 pharmacy students	Cross-sectional survey study, Likert scale questions, no control/comparator group included	Majority of students preferred onsite exams; cheating is a major concern associated with remote exams
9. Al-Alami, 2021, Jordan [40]	Examine student experiences with an online anatomy course	442 pharmacy students	End of class/activity survey, Likert scale and open-ended questions followed by thematic analysis, no control/comparator group included	Most students had positive perceptions of the course, noting they enjoyed flexibility but experienced technical challenges and missed face to face interactions
10. Jaam, 2021, Jordan [41]	Examination of perceptions of online assessments	17 (12 pharmacy students, 5 faculty members)	Post course survey analysis, one-to-one structured interviews followed by thematic analysis, no control/comparator group included	Lack of adequate communication, including receiving adequate feedback, were identified as key concerns
11. Cor, 2021, Canada [42]	Determination of the impact of open book exams on final exam scores and characteristics	262 pharmacy students (131 for each year)	Course performance assessment; pre-pandemic (closed book exams) versus during pandemic (open book exams) cohort study (data collected in different years)	An increased number of students passed open book exams but midterm:final exam score ratios remained unchanged
12. Nagy, 2021, Canada [43]	Examination of how student learning was impacted by COVID-19	397 pharmacy students	Survey study, open ended questions followed by thematic analysis, no control/comparator group included	Two main themes were identified relating to concerns; remote learning and mental health
13. Alzubaidi, 2021, International study [44]	Exploration of experiences and challenges of distance education during the COVID-19 pandemic	Administrators from 46 colleges of pharmacy	Cross-sectional survey study, Likert scale questions, no control/comparator group included	The majority of programs transitioned to online learning, were able to offer adequate support to faculty members. Assuring exam integrity and delivery of lab-based classes were key concerns
14. Hussain, 2021, USA [45]	Examination of student readiness, reception, and performance in an online versus in-person communications course	57 pharmacy students (25 in-person, 32 online)	Survey to assess student perception of readiness and performance pre- versus post-course, Likert scale questions	Remote learning did not impact pharmacy student self-perceived readiness or performance
15. Liu, 2021, Australia [46]	Examination of student challenges associated with COVID-19-driven curricular changes	774 pharmacy students	Review of student personalized learning plans (PLP) followed by thematic analysis; no control/comparator group included	Challenges identified included communication barriers, using new technology, time management, and negative emotional responses
16. Bartolo, 2020, Malta [47]	Examination of student perceptions of online learning and preparedness	75 (60 pharmacy students, 15 faculty)	Cross-sectional survey study, Likert scale questions, no control/comparator group included	Majority of students and faculty thought the transition to online learning was easy/they were prepared, students enjoyed flexibility but felt more alone
17. Al-Naimi, 2020, Qatar [32]	Examine student leader perceptions of pharmacy education during the pandemic	5 pharmacy students	Cross-sectional survey study, open-ended questions followed by thematic analysis, no control/comparator group included	Self-directed learning was noted as a key strength, increased study load was noted as a key weakness

* Some studies included participants from other healthcare professions; in these cases, only the number of participants from pharmacy programs and subgroup analysis data are reported in this table.

**Table 4:** Domain 4, student well-being (n = 14).

First Author, Year, Country	Study Objectives: Topic/Innovation/Intervention	Number of Participants	Study Design	Study Results
1. Hettinger, 2022, USA [11]	Comparison of stress levels for a skills-based lab courses pre-pandemic versus during the pandemic	801 pharmacy students (426 pre-pandemic, 375 during pandemic)	Cross-sectional survey study, Likert scale and open-ended questions followed by thematic analysis, pre- and post-pandemic	Stress levels decreased when skills-based labs were offered during the pandemic
2. Attarabeen, 2021, USA [48]	Determination of whether the pandemic increased student stress levels	258 pharmacy students (192 before pandemic, 66 during pandemic)	Cross-sectional survey study, Likert scale questions, pre- and post-pandemic	Student stress levels did not increase during the pandemic
3. Cernasev, 2021, USA [49]	Examination of the impact of COVID-19 on students	421 pharmacy students	Cross-sectional survey study, Likert scale and open-ended questions followed by thematic analysis, no control/comparator group included	Well-being and mental health struggles as well as stress were identified as being prevalent
4. Fuentes, 2021, USA [50]	Investigation of the coping, resilience, personal characteristics, and health behaviors on emotional well-being during the pandemic	286 students	Cross-sectional survey study, Likert scale questions, no control/comparator group included	Greater use of coping strategies and higher levels of resilience were significant predictors of increased emotional well-being
5. Hagemeier, 2021, USA [51]	Characterization of the impact of COVID-19 on student well-being	74 students	Cross-sectional survey study pre- versus post-transition to COVID-19-driven curricular changes (different years and different cohorts), Likert scale questions	Perceived overall well-being significantly decreased following implementation of COVID-19-driven curricular changes
6. Imeri, 2021, USA [52]	Exploration of the impact of the pandemic on wellness, challenges faced	13 pharmacy students	Semi-structured interviews followed by thematic analysis; no control/comparator group included	Stress levels were higher; contributing factors included limited social support, lack of collaborative work, challenging work requirements, electronic communications
7. Almhdawi, 2021, Jordan [53]	Investigation of students’ quality of life and its predictors during COVID-19	29 pharmacy students	Cross-sectional surveys, including Likert scale questions relating to learning as well as validated mental health surveys (DASS, SF12, IPAQ), no control/comparator group included	A significant number of students reported low quality of life and this correlated with several factors, including depression, stress, and IPAQ score
8. Al-Qerem, 2021, Jordan [54]	Evaluation of factors associated with anxiety and depression among pharmacy students	1085 pharmacy students	Cross-sectional survey study using BDI-II and STAI surveys, no control/comparator group included	A significant number of students have experienced anxiety and depression during the COVID-19 pandemic
9. Alomar, 2021, United Arab Emirates [55]	Assessment of perceived stress levels of quality of life during COVID-19	81 pharmacy students	Cross-sectional survey study; perceived stress scale and WHOQOL-BREF surveys, no control/comparator group included	Many students suffered a moderate amount of stress and experienced negative feelings, minimal impact on quality of life was observed
10. Alrasheedy, 2021, Saudi Arabia [56]	Assessment of the psychological impact of COVID-19 on students	232 pharmacy students	Cross-sectional survey study, Likert scale questions, no control/comparator group included	A significant number of students reported that they always or frequently felt anxious or nervous during the pandemic
11. Liu, 2021, Australia [46]	Characterization of students’ learning, well-being, and resilience during COVID-19	774 pharmacy students	Thematic review of responses to prompted questions, no control/comparator group included	The most coded challenges were ‘negative emotional response’ and ‘communication barriers during learning’, the most coded coping strategies were ‘using new technology’ and ‘time management’
12. Moreno-Fernandez, 2020, Spain [57]	Establishment of the impact of offering emotional intelligence workshops	47 pharmacy students	Survey before versus after an emotional intelligence workshop, Likert scale questions, pre- post-intervention study	Fewer students reported exhaustion, cynicism, and the feeling of ineffectiveness after attending the emotional intelligence workshops
13. Nagy, 2021, Canada [43]	Understanding of how student learning was impacted by COVID-19	397 pharmacy students	Cross-sectional survey, open ended questions followed by thematic analysis, no control/comparator group included	Mental health was identified as a key theme
14. Al-Naimi, 2020, Qatar [32]	Examine student leader perceptions of pharmacy education during the pandemic	5 pharmacy students	Cross-sectional survey study, open-ended questions followed by thematic analysis, no control/comparator group included	Student mental health and well-being was found to be the second highest challenge following postponement of experiential learning

## Data Availability

The data presented in this study are available in the Supplementary Materials section.

## Supplementary Materials

The following supporting information can be downloaded at: https://www.mdpi.com/article/10.3390/pharmacy10030060/s1.
