# Supplementary material for: COVID-19-Driven Improvements and Innovations in Pharmacy Education: A Scoping Review
Source: Pharmacy (Basel). 2022 Jun 4;10(3):60. doi: 10.3390/pharmacy10030060 (PMC9227261; doi:10.3390/pharmacy10030060)
Supplement: Supplementary file 1 [file pharmacy-10-00060-s001.zip › pharmacy-1687525-supplementary.pdf]

Supplementary Material: Executed search strategy, number of citations found for each search

Search for Pubmed, n = 384 articles

(("covid-19"[Title/Abstract] OR "covid19"[Title/Abstract] OR "covid-19"[Title/Abstract] OR "sars-cov-2"[Title/Abstract] OR "sars-cov2"[Title/Abstract] OR "2019-ncov"[Title/Abstract] OR "2019ncov"[Title/Abstract] OR "coronavirus"[Title/Abstract]) AND (("education"[Title/Abstract] OR "student*"[Title/Abstract] OR "trainee*"[Title/Abstract] OR "intern*"[Title/Abstract] OR "training*"[Title/Abstract] OR "rotation*"[Title/Abstract] OR "internship*"[Title/Abstract] OR "educator*"[Title/Abstract] OR "professor*"[Title/Abstract] OR "preceptor*"[Title/Abstract] OR "mentor*"[Title/Abstract] OR "instructor*"[Title/Abstract] OR "teacher*"[Title/Abstract]) AND ("pharmacy"[Title/Abstract] AND (2019:2022[pdat])

Search for Web of Science Core Collection, n = 538 articles

((TS=(covid-19 or COVID19 or COVID-19 or SARS-CoV-2 or SARS-CoV2 or 2019-nCoV or 2019nCoV or coronavirus)) AND TS=(pharmacy or pharmacist)) AND TS=(school or college or education or student or trainee or intern or training or internship or preceptor or educator or mentor or instructor or teacher)

Search for Ovid Medline, n = 126 articles

1 (covid-19 or COVID19 or COVID-19 or SARS-CoV-2 or SARS-CoV2 or 2019-nCoV or 2019nCoV or coronavirus).tw,kf.
2 exp coronavirus infections/
3 exp coronavirinae/
4 1 or 2 or 3
5 exp pharmacy education/
6 ((pharmacy or pharmacist*) adj3 (school* or education* or student* or trainee* or rotation* or training* or professor* or preceptor* or educator* or mentor* or instructor* or teacher*)).tw,kf.
7 5 or 6
8 4 and 7
9 limit 8 to yr="2019 -Current"

Search for MedEdPortal, n = 9 articles

(covid-19 OR coronavirus) AND pharmacy education
